# Gastrointestinal Failure score in critically ill patients: a prospective observational study

**DOI:** 10.1186/cc6958

**Published:** 2008-07-14

**Authors:** Annika Reintam, Pille Parm, Reet Kitus, Joel Starkopf, Hartmut Kern

**Affiliations:** 1 Clinic of Anaesthesiology and Intensive Care, University of Tartu, Puusepa, Tartu 51014, Estonia; 2 Department of Anaesthesiology and Intensive Care, East Tallinn Central Hospital, Ravi, Tallinn 10138, Estonia; 3 Clinic of Anaesthesiology and Intensive Care, Tartu University Hospital, Puusepa, Tartu 51014, Estonia; 4 Klinik für Anästhesiologie und Intensivmedizin, DRK Kliniken Berlin Köpenick, Salvador-Allende-Straße, Berlin 12559, Germany

## Abstract

**Introduction:**

There are no universally accepted diagnostic criteria for gastrointestinal failure in critically ill patients. In the present study we tested whether the occurrence of food intolerance (FI) and intra-abdominal hypertension (IAH), combined in a 5-grade scoring system for assessment of gastrointestinal function (the Gastrointestinal Failure [GIF] score), predicts mortality. The prognostic value of the GIF score alone and in combination with the Sequential Organ Failure Assessment (SOFA) score is evaluated, and the incidence and outcome of gastrointestinal failure is described relative to the GIF score.

**Methods:**

A total of 264 subsequently hospitalized patients, who were mechanically ventilated on admission and stayed in the intensive care unit (ICU) for longer than 24 hours, were prospectively studied. GIF score was documented daily as follows: 0 = normal gastrointestinal function; 1 = enteral feeding with under 50% of calculated needs or no feeding 3 days after abdominal surgery; 2 = FI or IAH; 3 = FI and IAH; and 4 = abdominal compartment syndrome (ACS). Admission parameters and mean GIF and SOFA scores for the first 3 days were used to predict ICU outcome.

**Results:**

FI developed in 58.3%, IAH in 27.3%, and both together in 22.7% of patients. The mean GIF score for the first 3 days in the ICU was identified as an independent risk factor for mortality (odds ratio = 3.02, 95% confidence interval = 1.63 to 5.59; P < 0.001). The GIF score integrated into the SOFA score allowed better prediction of ICU mortality than did the SOFA score alone, and was an independent predictor of mortality (odds ratio = 1.49, 95% confidence interval = 1.28 to 1.74; P < 0.001). The development of gastrointestinal failure (FI plus IAH) was associated with significantly higher ICU and 90-day mortality.

**Conclusion:**

The GIF score is useful for classifying information on the gastrointestinal system. The mean GIF score during the first 3 days in the ICU had high prognostic value for ICU mortality. Development of gastrointestinal failure is associated with significantly impaired outcome.

## Introduction

Gastrointestinal problems occur frequently and are associated with adverse outcomes in critically ill patients [1-4]. Despite this, there is no consensual means for obtaining a precise assessment of gastrointestinal function. Furthermore, gastrointestinal function is not included in any of the scoring systems widely used to assess organ failure in critical illness. That the importance of gastrointestinal failure in critically ill patients is underestimated is clear from its lack of clear definition. Numerous, mostly primary diagnosis-based definitions have been used by various investigators, making comparative interpretation of studies of gastrointestinal function rather difficult, if not impossible [5].

More than 10 years ago the summary of a roundtable conference in gut dysfunction in critical illness [6] concluded that intestinal function is an important determinant of outcome in critically ill patients; that there is no objective, clinically relevant definition of intestinal dysfunction in critical illness; and that any definition developed in the future should grade the severity of the dysfunction. A scoring system for gastrointestinal dysfunction is thus warranted, and the continuing lack of a systamtic approach is limiting studies conducted to assess epidemiology, time course, risk factors and treatment.

Different gastrointestinal complications (decreased bowel sounds, delayed gastric emptying, and diarrhoea) may occur in up to 50% of mechanically ventilated patients [1,7]. Intolerance to gastric feeding due to delayed gastric emptying occurs in approximately half of critically ill patients [2,7-10] and has an adverse impact on intensive care unit (ICU) mortality and length of stay [2,7,9].

Monitoring of intra-abdominal pressure (IAP) is gaining greater popularity in everyday clinical practice. It is easily performed and yield a reliable value that may be interpreted. Several studies have demonstrated the impact that intra-abdominal hypertension (IAH) has on mortality [11-13]. However, IAP has not been proven to represent an adequate measure of gastrointestinal function, and there is some evidence suggesting that not all patients with IAH have gastrointestinal problems and *vice versa *[14].

Based on the findings described above, we hypothesized that a combination of IAP and gastrointestinal symptoms might be a good basis for evaluating gastrointestinal dysfunction in critically ill patients. With the goal of developing a scoring system for gastrointestinal failure, we combined gastrointestinal symptoms and IAH into a 5-grade scale – the Gastrointestinal Failure (GIF) score. Similar to other organ failure scores, the instrument requires validation within the setting of an assessment of mortality among patients with different GIF scores.

The aim of the present study was to test the accuracy of the GIF score. We examined GIF as a part of multiple organ failure in a population of mixed ICU patients by evaluating the prognostic value of the GIF score both alone and in combination with the Sequential Organ Failure Assessment (SOFA) score. We also aimed to describe the incidence and outcome of gastrointestinal failure according to GIF score.

## Materials and methods

All mechanically ventilated patients subsequently admitted to the mixed surgical-medical ICU of Tartu University Hospital from September 2006 to September 2007 were screened for inclusion in the present prospective study. Patients treated for at least 24 hours were included in further analyses. On admission, the following parameters were recorded: age, sex, body mass index, readmission rate, diabetes, Acute Physiology and Chronic Health Evaluation (APACHE II) score [15], surgical profile, and whether laparatomy was performed (immediately before ICU admission or during the first 24 hours). The SOFA score [16], mean arterial pressure, central venous pressure, peak inspiratory pressure, positive end-expiratory pressure, IAP, lactate, fluid gain, use of vasopressor/inotrope and sedation were recorded on daily basis. Gastrointestinal function of the patients was assessed daily using the GIF score, described in Table 1.

**Table 1:** GIF score

Points	Clinical symptomatology
0	Normal gastrointestinal function
1	Enteral feeding <50% of calculated needs or no feeding 3 days after abdominal surgery
2	Food intolerance (enteral feeding not applicable due to high gastric aspirate volume, vomiting, bowel distension, or severe diarrhoea) or IAH
3	Food intolerance and IAH
4	Abdominal compartment syndrome

GIF, Gastrointestinal Failure; IAH, intra-abdominal hypertension.

Enteral feeding was started as early as possible, but not within the first days after major abdominal surgery. Food intolerance (FI) was diagnosed when applied enteral feeding appeared to be unsuccessful and had to be discontinued because of repeated or profuse vomiting, high gastric residuals, ileus, severe diarrhoea, abdominal pain, or distension. FI was not registered when the patient was electively not fed during the first 3 days after laparatomy. Gastric residual volume was considered to be high when it exceeded the volume previously given enterally.

IAP was measured via the bladder, with patients in the supine position, using the closed loop system repeated measurements technique [17]. The IAP was measured at least twice a day when normal values were recorded, and at least four times a day if IAP was found to be elevated above 12 mmHg. Mean and maximum values of IAP were documented daily. Mean IAP was used to calculate daily GIF score. IAH was defined as an IAP that was persistently 12 mmHg or greater [18]. Abdominal compartment syndrome was defined as an IAP that was persistently above 20 mmHg, along with onset of a new organ failure. Gastrointestinal failure was considered to be present when IAH and FI occurred simultaneously.

ICU, 28-day and 90-day mortality, and durations of ICU stay and mechanical ventilation were primary outcome parameters. The SOFA+GIF score was calculated each day by summarizing the SOFA score and the GIF score of the respective day in each patient.

The Ethics Committee of the University of Tartu approved the study. Written informed consent was not considered necessary for the study, because it was observational in nature. No special interventions were applied. All of the data were rendered anonymous before analysis, and no harm resulted from the study that could be weighed against benefit.

### Statistical analysis

Statistical Package for the Social Sciences (version 15.0; SPSS Inc., Chicago, IL, USA) software was used for statistical analysis. *t*-test for continuous variables and c^2 ^test for categorical variables were used for comparisons of two groups. Mean scores during the first 3 days were calculated as a mean of individual values for 3 days for every patient. Univariate analyses of admission parameters were applied to identify risk factors for ICU mortality. Parameters with *P *< 0.2 were thereafter entered into the multiple logistic regression model to identify independent risk factors. The means of the variables for the first 3 days were thereafter added to the admission parameters in multiple regression analysis. The first day values of the parameters, included in the scores, were removed from this analysis to exclude coupling. Receiver operating characteristic curves were used to determine the likelihood ratios for the abilities of the GIF score, SOFA score and SOFA+GIF score to predict ICU mortality. Kaplan-Meier curves and log-rank tests were used to compare survival between patients with and those without gastrointestinal failure. Data are presented as mean (standard deviation), if not stated otherwise. *P *< 0.05 was considered statstically significant.

## Results

A total of 373 patients were treated in the general ICU of Tartu University Hospital during the study period; 264 patients were receiving mechanical ventilation at admission and stayed in the ICU for at least 24 hours, and were therefore included in further analysis.

Of these patients, 93.9% were admitted on an emergent basis. The case-mix does not include cardiac surgical or neurosurgical patients. Most of the surgical patients were admitted because of respiratory failure (43%) or shock (29%). Among medical patients, the main causes for admission were coma (30%), shock (21%), postresuscitation state (20%) and respiratory failure (12%). Admission parameters and outcome data for the included patients are presented in Table 2.

**Table 2:** Admission and outcome parameters of study patients

Admission parameters		IAH			FI		
	Total	Absent	Present	*P*	Absent	Present	*P*
Number of patients (%)	264 ± 100.0	192 ± 72.3	72 ± 27.3		110 ± 41.7	154 ± 58.3	
Male sex (*n *[%])	166 ± 62.9	117 ± 60.9	49 ± 68.1	0.178	60 ± 54.5	106 ± 68.8	0.013
Age (years)	53.8 ± 20.0	52.3 ± 21.0	57.8 ± 16.8	0.047	48.6 ± 21.0	57.5 ± 18.5	<0.001
Diabetes (*n *[%])	35 ± 13.3	26 ± 13.5	9 ± 12.5	0.502	11 ± 10.0	24 ± 15.6	0.128
Readmission (*n *[%])	8 ± 3.0	6 ± 3.1	2 ± 2.8	0.622	2 ± 1.8	6 ± 3.9	0.287
Sedation (*n *[%])	243 ± 92.0	174 ± 90.6	69 ± 95.8	0.125	98 ± 89.1	145 ± 94.2	0.103
Vasoactive/inotrope (*n *[%])	200 ± 75.8	136 ± 70.8	64 ± 88.9	0.001	67 ± 60.9	133 ± 86.4	<0.001
Surgical profile (*n *[%])	175 ± 66.3	122 ± 63.5	53 ± 73.7	0.080	67 ± 60.9	108 ± 70.1	0.077
Laparatomy (*n *[%])	60 ± 23.3	21 ± 15.6	31 ± 43.7	<0.001	9 ± 8.4	51 ± 34.0	<0.001
Enteral feeding (*n *[%])	49 ± 18.6	42 ± 21.9	7 ± 9.7	0.015	25 ± 22.7	24 ± 15.6	0.095
APACHE II score (points)	14.2 ± 7.7	13.5 ± 7.6	16.0 ± 7.7	0.016	11.8 ± 7.3	15.8 ± 7.6	<0.001
BMI (kg/m^2^)	27.6 ± 13.1	25.4 ± 4.6	33.1 ± 22.2	<0.001	25.8 ± 5.6	28.9 ± 16.4	0.080
SOFA score (points)	7.0 ± 4.2	6.3 ± 4.2	8.9 ± 3.6	<0.001	5.3 ± 4.2	8.2 ± 3.8	<0.001
Fluid gain in first 24 hours (l)	2.4 ± 3.6	2.0 ± 2.4	3.6 ± 5.5	0.001	1.7 ± 2.4	2.9 ± 4.2	0.007
MAP (mmHg)	81.5 ± 15.9	82.1 ± 16.4	80.5 ± 15.0	0.512	82.1 ± 15.8	81.3 ± 16.1	0.758
IAP (mmHg)	8.5 ± 4.7	6.5 ± 3.8	12.1 ± 4.0	<0.001	6.3 ± 4.0	9.5 ± 4.6	<0.001
CVP (mmHg)	11.6 ± 5.7	10.3 ± 4.9	14.3 ± 6.3	<0.001	10.4 ± 5.1	12.2 ± 5.9	0.031
PIP (cmH_2_O)	23.9 ± 6.2	22.6 ± 6.0	27.2 ± 5.2	<0.001	22.5 ± 6.5	24.8 ± 5.7	0.006
PEEP (cmH_2_O)	9.2 ± 4.2	8.5 ± 4.1	11.2 ± 3.7	<0.001	7.8 ± 4.1	10.2 ± 3.9	<0.001
Lactate (mmol/l)	4.6 ± 5.4	4.4 ± 4.6	4.9 ± 6.9	0.519	4.4 ± 4.9	4.7 ± 5.7	0.741
FI (*n *[%])	124 ± 47.0	69 ± 35.9	55 ± 76.4	<0.001	-	-	
IAH (*n *[%])	42 ± 15.9	-	-		3 ± 2.7	39 ± 25.3	<0.001
Gastrointestinal failure (FI+IAH; *n *[%])	36 ± 13.6	-	36 ± 50.0		-	36 ± 23.4	
Outcome parameters							
MV (days)	7.4 ± 11.9	4.3 ± 6.1	15.5 ± 18.0	<0.001	3.6 ± 5.9	10.0 ± 14.1	<0.001
ICU (days)	8.8 ± 12.8	5.6 ± 7.3	17.5 ± 19.0	<0.001	4.8 ± 6.9	11.7 ± 15.1	<0.001
ICU mortality (*n *[%])	39 ± 14.8	21 ± 10.9	18 ± 25.0	0.005	7 ± 6.4	32 ± 20.8	0.001
28-day mortality (*n *[%])	54 ± 20.5%	36 ± 18.8%	18 ± 25.0%	0.171	13 ± 11.8%	41 ± 26.6%	<0.001
90-day mortality (*n *[%])	75 ± 28.4%	48 ± 25.0%	27 ± 37.5%	0.033	17 ± 15.5%	58 ± 37.7%	0.002

The values are presented as mean ± standard deviation. APACHE, Acute Physiology and Chronic Health Evaluation; BMI, body mass index; CVP, central venous pressure; FI, food intolerance; IAH, intra-abdominal hypertension; IAP, intra-abdominal pressure; ICU, intensive care unit; MAP, mean arterial pressure; MV, mechanical ventilation; PEEP, positive end-expiratory pressure; PIP, peak inspiratory pressure; SOFA, Sequential Organ Failure Assessment.

### Incidence of food intolerance and intra-abdominal hypertension

FI was observed in 154 patients (58.3%), and it developed predominantly during the first 3 days of admission (144/154 [93.5%]). Seventy-two patients (27.3%) developed IAH, and five of them (6.9% of IAH patients) suffered from ACS. Of IAH patients, 63 out of 72 (87.5%) developed the syndrome during their first 3 days in the ICU.

Gastrointestinal failure (FI plus IAH) developed in 60 patients (22.7%); in 36 of them (13.6% of the study population) it was already present on the day of admission (Table 2).

### Patient management

Metoclopramide was routinely used as a prokinetic drug in case of feeding problems. All patients suffering from IAH received at least one of the noninvasive techniques suggested to decrease IAP [19]: paracentesis, continuous venovenous haemodiafiltration, laxatives, nasogastric aspiration, sedation, negative fluid balance, relaxants, rectal gas tube, or enema. Laparatomy was performed in three patients with ACS during the first 2 days; one of them (a trauma patient) survived, and two patients (pancreatitis as the primary disease) died. Both patients with ACS who did not undergo laparatomy died.

In 92% of patients, sedation was started upon admission or previously instituted sedation was continued in the ICU; 76% of patients received opiods. Enteral feeding was successfully applied in only 18.6% of patients on their admission day; 47% of patients did not tolerate enteral feeding; and feeding was not started in 34.4% of patients on admission.

### GIF score

The GIF score was documented overall in 2,348 patient-days. GIF score 0 was observed in 52.0%, 1 in 12.2%, 2 in 27.8%, 3 in 7.7%, and 4 in 0.3% of days. Jejunal feeding was applied in 11% of all patient-days, but very rarely (1%) during the first 3 days. Of the patients who had a GIF score of 1 for 1 day, 27.6% developed higher GIF scores later. In patients who had a GIF score of 1 for 2 or more subsequent days, progression of the syndrome was more common; 72.3% of them subsequently developed higher GIF scores during the following days. The mean ± standard deviation GIF score during first 3 days in the ICU was 1.2 ± 0.9 points, being significantly different between survivors and nonsurvivors (1.1 ± 0.8 versus 2.0 ± 1.0, respectively; *P *< 0.001). The mean of the maximum GIF score was 1.6 ± 1.0 in survivors versus 2.3 ± 1.1 in nonsurvivors (*P *< 0.001).

### Outcome

ICU mortality in the study population was 14.8%. At 28 and 90 days the mortality rates were 20.5% and 28.4%, respectively. The lengths of ICU stay and mechanical ventilation, and ICU and 90-day mortality rates were significantly different between IAH and non-IAH patients, as well as between FI and non-FI patients (Table 2).

**Figure 1:**
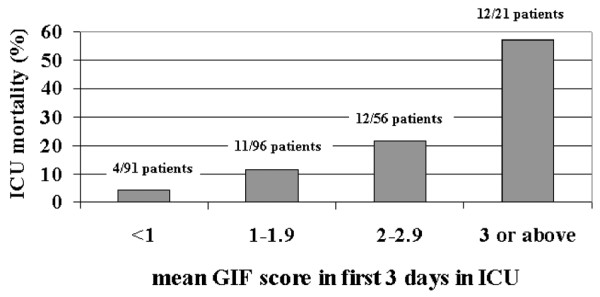
ICU mortality of patients according to their mean GIF score. ICU, intensive care unit; GIF, Gastrointestinal Failure (score).

A high mean GIF score during the first 3 days of the ICU stay was associated with a high rate of mortality (Figure 1). The patients with gastrointestinal failure (simultaneous occurrence of IAH and FI) suffered from an ICU mortality of 28.1%, as compared with 10.8% in patients without this syndrome (*P *= 0.001). The mortality was also higher after 90 days (40.0% versus 25.0%; *P *= 0.019), but not after 28 days (28.3% versus 18.1%; *P *= 0.065).

### Prediction of outcome

#### Admission parameters and prediction of ICU mortality

In multiple regression analysis, only two admission parameters (SOFA and fluid balance during the first 24 hours) were identified as independent predictors of ICU mortality in the study population.

#### Means of the 3 three days in combination with admission parameters

As expected, the mean SOFA score for the first 3 days was a better predictor than its value on the first day (odds ratio [OR] = 1.40, 95% confidence interval [CI] = 1.18 to 1.64 [*P *< 0.001] versus OR = 1.36, 95% CI = 1.02 to 1.82 [*P *= 0.037]). The cumulative fluid balance, mean IAP and development of FI (yes/no) during the first 3 days had significant impacts in univariate analyses, but they were not identified as independent risk factors for mortality.

The mean GIF score during the first 3 days (used instead of the mean IAP and development of FI) was identified as an independent risk factor for ICU mortality (OR = 3.02, 95% CI = 1.63 to 5.59; *P *< 0.001). The mean SOFA+GIF score for the first 3 days demonstrated slightly better prediction of ICU mortality than did the SOFA score alone (OR = 1.49, 95% CI = 1.28 to 1.74; *P *< 0.001).

#### Combination of GIF and SOFA scores

**Figure 2:**
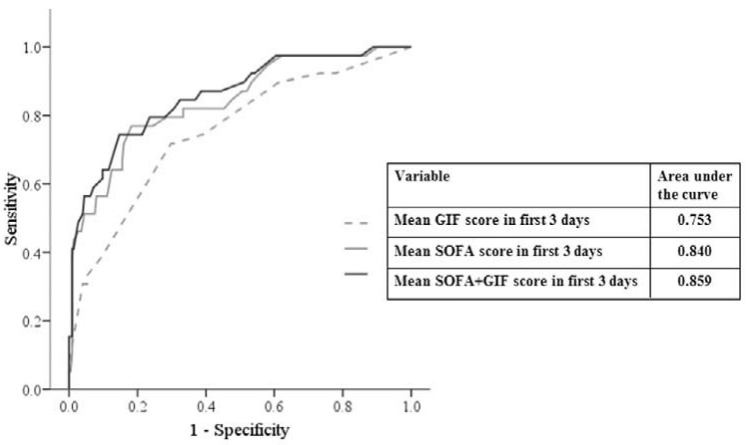
ROC curves with different scores in prediction of ICU mortality. ICU, intensive care unit; ROC, receiver operating characteristic.

The combination of mean SOFA and GIF score during the first 3 days exhibited the greatest area under the curve (0.895), being superior to those for the mean SOFA score (0.840) and the mean GIF score (0.753) alone (Figure 2). In the regression analysis for prediction of ICU mortality (Table 3), the GIF score for first 3 days had the second highest OR (2.20, 96% CI = 1.28–3.78; *P *= 0.004), after the cardiovascular SOFA subscore (OR = 5.91, CI = 2.83 to 12.33; *P *< 0.001).

**Table 3:** SOFA subscores and GIF score in regression analysis for prediction of ICU mortality

Score/subscore	*P*	OR	95% CI
Cardiovascular SOFA	<0.001	5.91	2.83–12.33
GIF score	0.004	2.20	1.28–3.78
Hepatic SOFA	0.024	1.75	1.075–2.86
Renal SOFA	0.087	1.39	0.95–2.04
Central nervous system SOFA	0.159	1.23	0.92–1.65
Haematological SOFA	0.712	0.92	0.57–1.47
Respiratory SOFA	0.518	0.84	0.48–1.44

CI, confidence interval; GIF, Gastrointestinal Failure (score); OR, odds ratio; SOFA, Sequential Organ Failure Assessment.

#### Gastrointestinal failure (intra-abdominal hypertension plus food intolerance) and outcome

**Figure 3:**
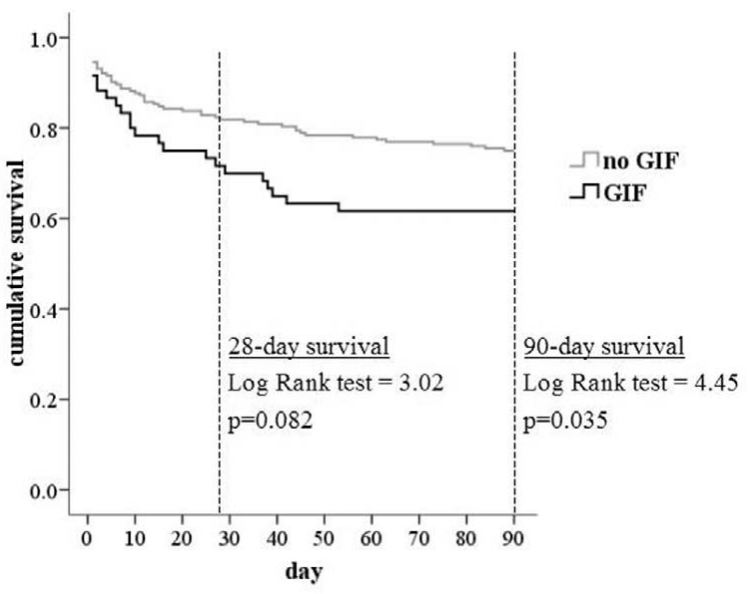
Survival: absence versus presence of gastrointestinal failure. Shown is the cumulative survival of patients without gastrointestinal failure (maximum Gastrointestinal Failure [GIF] score during intensive care unit (ICU) stay ≤ 2) versus patients with gastrointestinal failure (maximum GIF score during ICU stay of 3 or 4).

The 90-day cumulative survival of patients with gastrointestinal failure was significantly impaired in comparison with patients without gastrointestinal failure (log-rank test = 4.45; *P *= 0.035). There was no significant difference in 28-day survival, as shown in Figure 3. Ten patients with gastrointestinal failure (15.6%) were still in the ICU on day 28, and three of them died in the ICU, whereas none of the patients without GIF died in the ICU after day 28.

## Discussion

This single-centre pilot study demonstrates the usefulness of the GIF score – a combined assessment of FI and IAP – for dynamic assessment of gastrointestinal function in critically ill patients. Combining FI with IAP values appeared to be a better predictor of outcome than either of these parameters alone. The mean GIF score of the first 3 days is an independent risk factor for ICU mortality. Furthermore, the score may add predictive power to the SOFA score in outcome prediction.

Gastrointestinal function was demonstrated to influence the ICU outcome in previous studies. However, the absence of a scaled system for assessing gastrointestinal function has been a major limiting factor in these studies. The role of the gastrointestinal tract as a motor of multiple organ failure was identified more than 2 decades ago and was more recently confirmed by Clark and Coopersmith [20]. Nevertheless, because of a lack of definition, diagnostic reliability [21,22] and accurate assessment of incidence, gastrointestinal failure is not included in severity of illness scoring systems currently in use.

About half of the patients of included in the present study developed FI during the first 3 days after ICU admission. These patients were significantly older and more severely ill (higher APACHE and SOFA scores). They also stayed longer in ICU and exhibited greater mortality than did patients with normal gastrointestinal function. The prevalence of FI was described to be within a similar range in the literature and has been shown to influence outcomes [2,7,8]. Mortality of food intolerant patients in the present study was lower than in previous studies, with mortality ranging between 30% to 40% [2,7], but also significantly higher than in patients without FI.

At first glance, it appears reasonable to use more specific gastrointestinal symptoms, such as bleeding, high gastric residual volumes and so on, when assessing gastrointestinal function. It should be noted, however, that there are a number of obstacles to their use in this setting: gastrointestinal bleeding occurs rarely [22,23]; the incidence of vomiting is influenced by nasogastric aspiration; and high gastric residual volume is not defined uniformly and exhibits only a weak correlation with gastric emptying [23]. None of these potential markers of gastrointestinal function take into account factors such as severe diarrhoea, which is often treated by reducing the rate of enteral feeding [1] and has been shown to double the likelihood of graft loss and patient death after kidney transplantation [24].

Most attempts to define gastrointestinal dysfunction described in the literature are based on diagnosis rather than function. For example, the presence of cholecystitis [25] and gastrointestinal bleeding [22,25] were previously suggested to identify GIF. Such approaches do not consider a functional assessment of the gastrointestinal tract – a highly complex organ. Surprising variability also exists in definitions of FI. Although most authors define it based on high gastric residuals or vomiting [2,9,23,26], others also include include abdominal pain or distension and diarrhoea as reasons to stop feeding and declare FI to be present [27]. Even though FI is a rather subjective variable, it is – in our opinion – the most universally used clinical characteristic of gastrointestinal failure, probably covering the entire spectrum of gastrointestinal symptoms. As has also been stated by the experts, despite obvious limitations to the definition of intolerance to enteral feeding, it provides a functional assessment with some clinical relevance [6].

IAH did not occur in our patients as frequently as FI; it developed in only one-third of them. These data are in accordance with observations from Malbrain and colleagues [28], who described a similar prevalence of IAH in a mixed ICU population. Various studies conducted in selected patients groups have identified an adverse impact of IAP on ICU outcome [13,29]. Malbrain and coworkers [11] found the development of IAH during the ICU stay, but not IAP on admission, to be an independent risk factor for mortality [11]. However, prediction of outcome based on events occurring during the entire ICU period is of somewhat limited value. Therefore, we limited our assessment to mean values during the first 3 days. Accordingly, the GIF score, but not IAH or FI, appeared to be an independent predictor of outcome.

Little is known about the combination of FI with IAH. Our data clearly demonstrate that patients manifesting these two signs are not fully overlapping groups; not all the patients with gastrointestinal problems have IAH and *vice versa*. Of the patients with IAH on admission, 76% also experienced FI, whereas only 25% the patients with FI had IAH. Some of the patients who subsequently developed IAH exhibited FI on admission, and only a few patients who went on to develop FI exhibited IAH on admission. This, in our opinion, further supports the need to combine these two variables in the GIF score. The definite strength of IAP measurement in this setting is its objective and reproducibly measurable numeric value.

It is difficult to estimate the extent to which the route of enteral feeding influences the GIF score. However, the advantage of post-pyloric versus gastric feeding with respect to outcome is not yet established [30], and thus the current evidence does not support routine use of post-pyloric feeding in the critically ill [31]. The post-pyloric route is probably not the most common choice during the first few days of intensive care, even though Montejo and coworkers [3] reported a lower incidence of gastrointestinal complications in patients receiving early jejunal nutrition. It may be speculated that enteral feeding itself produces an increase in IAP in critically ill patients, but we did not observe such an association in a preliminary study [32]. Feeding and sedation strategies are expected to influence gastrointestinal function. However, if we are to evaluate the impact that different treatments have, we require a tool for evaluating gastrointestinal dysfunction.

The main limitation of the present study is that only those patients with prolonged ICU stay (> 24 hours) were included. Patients treated in the ICU for less than 24 hours arguably contain a mixture of the least and most severely ill patients. This pre-selection may bias the results, which might account for the low predictive power of the APACHE II score. However, in most short-staying patients the IAH and FI are not usually the key issues of the treatment. IAH is seldom measured in patients who die within a few hours after ICU admission. Recognizing this delay in IAH monitoring, it is important to emphasize that in a few ACS patients prompt IAH measurement might be crucial to making the correct therapeutic decisions and thus maximizing survival. Parsak and coworkers [33] recently demonstrated an advantage of early surgical intervention in patients with primary ACS, and they emphasized the importance of controlling IAP during the early postoperative period. Although mortality from ACS remains high, the avoidance of this end-stage syndrome may be possible with IAP monitoring and treatment strategies recommended by the World Society of the Abdominal Compartment Syndrome [34].

The observed high predictive value of the mean SOFA score for ICU outcome is in accordance with the findings of several previous studies. The predictive power of the mean SOFA score during the first 3 days is correctly placed between the those of the mean SOFA score for the whole ICU period (OR = 3.06) and the SOFA score at 48 hours (OR = 1.45) [35]. Similar predictive value of SOFA subscores was observed in cardiac surgical patients [36]. The cardiovascular SOFA score appeared to be the most powerful, whereas the respiratory and haematological SOFA scores were the least powerful [36]. The excellent performance of the GIF score in this setting once more confirms the importance of gastrointestinal failure among other organ failures. The cumulative survival curves of patients with or without GIF further emphasize this finding. The fact that the difference in favour of patients without GIF is significant with respect to 90-day survival, but not survival at 28 days, is probably accounted for by the longer ICU stay with subsequently higher ICU mortality in GIF patients.

A limitation of this study is its single-centre design. The GIF score is probably influenced by both case-mix and treatment strategies; therefore, variation between centres may occur.

In our opinion, the major limitation of the GIF score is the subjectivity of estimation of the presence of FI. There is no consensual definition of FI, and variability in the definitions used in the literature is considerable. Also, continuity of the variables in the GIF score could be improved. The score is not exactly a continuum of alterations, as suggested for an organ failure score by Ferreira and coworkers [35]. However, it does fulfill the other criterion established by that author; specifically, it is based on easily accessible variables [35]. As the mean score of the first 3 days is not very helpful in everyday ICU practice, we propose a possible interpretation of the daily GIF score by reference to the RIFLE classification [37] as follows: Risk, GIF score 1 for at least 2 days; Injury, GIF score 2; Failure, GIF score 3; and End-stage, GIF score 4.

## Conclusion

The mean GIF score during the first 3 days on the ICU exhibited high prognostic value in terms of predicting ICU mortality. The GIF score is useful for categorizing information on the gastrointestinal system. Development of gastrointestinal failure during the ICU stay is associated with significantly higher ICU and 90-day mortality. Further multicentre studies should confirm whether the GIF score could be adopted as a useful subscore for gastrointestinal tract assessment in the SOFA score.

## Key messages

• Gastrointestinal failure is associated with adverse outcomes.

• Gastrointestinal failure is an important part of multiple organ failure.

• FI and IAH may be used together to evaluate the gastrointestinal system in critically ill patients.

• The GIF score is useful for categorizing information on the gastrointestinal system.

• The GIF score has high predictive value for ICU mortality.

## Abbreviations

ACS = abdominal compartment syndrome; APACHE = Acute Physiology and Chronic Health Evaluation; CI = confidence interval; FI = food intolerance; GIF = Gastrointestinal Failure (score); IAH = intra-abdominal hypertension; IAP = intra-abdominal pressure; ICU = intensive care unit; OR = odds ratio.

## Competing interests

The authors declare that they have no competing interests.

## Authors' contributions

AR participated in designing the study and statistical analysis, and drafted the manuscript. PP and RK conducted data collection and participated in the statistical analysis. JS participated in designing the study and writing of the manuscript. HK participated in designing the study and helped to draft the manuscript. All authors read and approved the final manuscript.
